# Optimal threshold estimation for binary classifiers using game theory

**DOI:** 10.12688/f1000research.10114.3

**Published:** 2017-02-08

**Authors:** Ignacio Enrique Sanchez

**Affiliations:** 1 Protein Physiology Laboratory, University of Buenos Aires, Buenos Aires, Argentina

**Keywords:** Binary classifier, ROC curve, accuracy, optimal threshold, optimal cutoff, class imbalance, game theory, minimax principle.

## Abstract

Many bioinformatics algorithms can be understood as binary classifiers. They are usually compared using the area under the receiver operating characteristic ( *ROC*) curve. On the other hand, choosing the best threshold for practical use is a complex task, due to uncertain and context-dependent skews in the abundance of positives in nature and in the yields/costs for correct/incorrect classification. We argue that considering a classifier as a player in a zero-sum game allows us to use the minimax principle from game theory to determine the optimal operating point. The proposed classifier threshold corresponds to the intersection between the *ROC* curve and the descending diagonal in *ROC* space and yields a minimax accuracy of 1-FPR. Our proposal can be readily implemented in practice, and reveals that the empirical condition for threshold estimation of “specificity equals sensitivity” maximizes robustness against uncertainties in the abundance of positives in nature and classification costs.

## Introduction

Many bioinformatics algorithms can be understood as binary classifiers, as they are used to investigate whether a query entity belongs to a certain class ^1^. Supervised learning trains the classifier by looking for the rule that gives the correct answers to a training set of question-answer pairs. On the other hand, score-based binary classifiers are trained in a non-supervised manner. Such classifiers use a scoring function that assigns a number to the query. During training, the scoring function is modified to give different scores to the positives and negatives in the training set. After training, the classifier is used by assigning a query to the class under consideration if its score surpasses a threshold. A minority of users is able to choose a threshold using their understanding of the algorithm, while the majority uses the default threshold.

**Figure 1.**
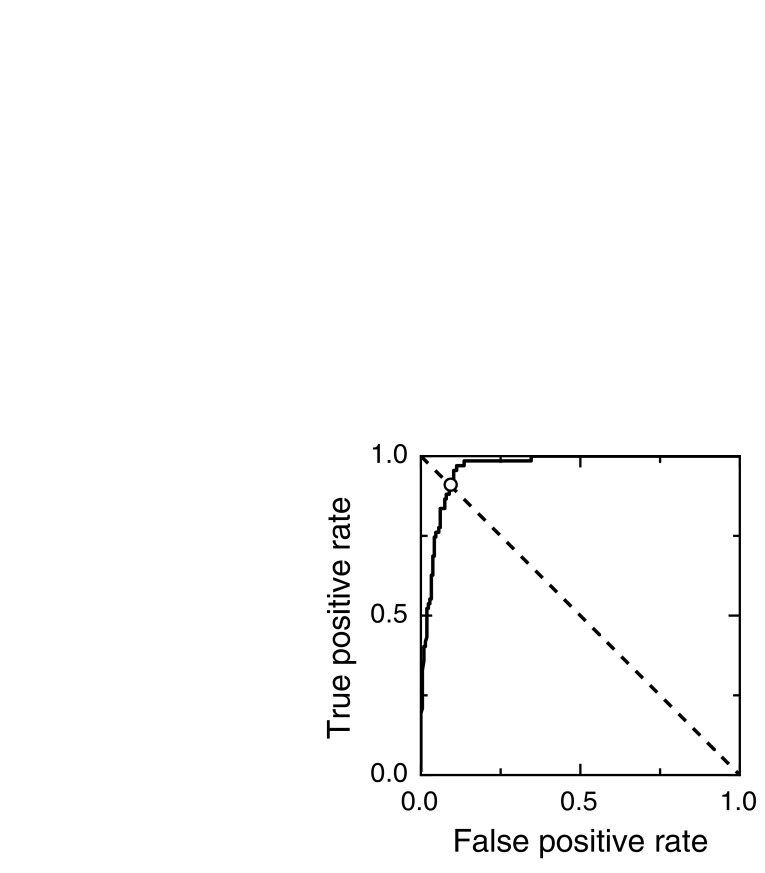
Optimal threshold estimation in *ROC* space for a binary classifier using game theory. The descending diagonal *TPR* = 1 – *FPR* (dashed line) minimizes classifier performance with respect to *q _P_*. The intersection between the receiver operating characteristic ( *ROC*) curve (continuous line) and this diagonal maximizes this minimal, worst-case utility and determines the optimal operating point according to the *minimax* principle (empty circle).

Binary classifiers are often compared under a unified framework, the receiver operating characteristic ( *ROC*) curve ^2^. Briefly, classifier output is first compared to a training set at all possible classification thresholds, yielding the confusion matrix with the number of true positives ( *TP*), false positives ( *FP*), true negatives ( *TN*) and false negatives ( *FN*) ( Table 1). The *ROC* curve plots the true positive rate ( *TPR* = *TP*/( *TP* + *FN*)), also called sensitivity,) against the false positive rate ( *FPR* = *FP*/( *FP* + *TN*)), which equals 1-specificity) ( Figure 1, continuous line). Classifier model selection often aims at maximizing the area under the *ROC* curve, which amounts to maximizing the probability that a randomly chosen positive is ranked before a randomly chosen negative ^2^. This summary statistic measures performance without committing to a threshold.

**Table 1.** Confusion matrix for training of a binary classifier. TP: Number of true positives. FP: Number of false positives. FN: Number of false negatives. TN: Number of true negatives.

		Training set	
		**p**	**n**
**Classifier output**	**p’**	*TP*	*FP*
	**n’**	*FN*	*TN*

Practical application of a classifier requires using a threshold-dependent performance measure to choose the operating point ^1, 3^. This is in practice a complex task because the application domain may be skewed in two ways ^4^. First, for many relevant bioinformatics problems the prevalence of positives in nature *q _P_* = ( *TP* + *FN*)/( *TP* + *TN* + *FP* + *FN*) = 1 - *q _N_* does not necessarily match the training set *q _P_* and is hard to estimate ^2, 5^. Second, the yields (or costs) for correct and incorrect classification of positives and negatives in the machine learning paradigm ( *Y _TP_*, *Y _TN_*, *Y _FP_*, *Y _FN_*) may be different from each other and highly context-dependent ^1, 3^. Points in the *ROC* plane with equal performance are connected by iso-yield lines with a slope, the skew ratio, which is the product of the class skew and the yield skew ^4^:

$$SKEW\;RATIO=\frac{q_{N}.(Y_{FP}+Y_{TN})}{q_{P}.(Y_{TP}+Y_{FN})}\;(1)$$

The skew ratio expresses the relative importance of negatives and positives, regardless of the source of the skew ^4^. Multiple threshold-dependent performance measures have been proposed and discussed in terms of skew sensitivity ^3, 4^, but often not justified from first principles.

## Theory

Game theory allows us to consider a binary classifier as a zero-sum game between nature and the classifier ^6^. In this game, nature is a player that uses a mixed strategy, with probabilities *q _P_* and *q _N_*=1- *q _P_* for positives and negatives, respectively. The algorithm is the second player, and each threshold value corresponds to a mixed strategy with probabilities *p _P_* and *p _N_* for positives and negatives. Two of the four outcomes of the game, *TP* and *TN*, favor the classifier, while the remaining two, *FP* and *FN*, favor nature. The game payoff matrix ( Table 2) displays the four possible outcomes and the corresponding classifier utilities *a*, *b*, *c* and *d*. The *Utility* of the classifier within the game is:

$$UTILITY=\frac{a.TP+d.TN+b.FP+c.FN}{TP+TN+FP+FN}\;(2)$$

**Table 2.** Payoff matrix for a zero-sum game between nature and a binary classifier. *a*: Player I utility for a true positive. *b*: Player I utility for a false positive. *c*: Player I utility for a false negative. *d*: Player I utility for a true negative.

		**Player II: Nature**	
		**p**	**n**
**Player I: Classifier**	**p’**	*a*	*b*
	**n’**	*c*	*d*

The payoff matrix for this zero-sum game corresponds directly to the confusion matrix for the classifier, and the game utilities *a*, *b*, *c*, *d* correspond to the machine learning yields *Y _TP_*, *Y _FP_*, *Y _FN_*, *Y _TN_*, respectively ( Table 1). In our definition of the skew ratio, the uncertainty in the values of *a*, *b*, *c* and *d* is equivalent to the uncertainty in the values of *q _P_* and *q _N_* ^4^. Thus, we can study the case a=d=1 and b=c=0 without loss of generality ^4^. Classifier *Utility* within the game then reduces to the *Accuracy* or fraction of correct predictions ^2– 4^. In sum, maximizing the *Utility* of a binary classifier in a zero-sum game against nature is equivalent to maximizing its *Accuracy*, a common threshold-dependent performance measure.

We can now use the *minimax* principle from game theory ^6^ to choose the operating point for the classifier. This principle maximizes utility for a player within a game using a pessimistic approach. For each possible action a player can take, we calculate a worst-case utility by assuming that the other player will take the action that gives them the highest utility (and the player of interest the lowest). The player of interest should take the action that maximizes this minimal, worst-case utility. Thus, the *minimax* utility of a player is the largest value that the player can be sure to get regardless of the actions of the other player. In our case, nature is not a conscious player that chooses the action that gives them the highest utility. We instead understand our application of the minimax principle as the consideration of a worst-case scenario for the skew ratio.

In our classifier *versus* nature game, *Utility/Accuracy* of the classifier is skew-sensitive, depending on *q _P_* for a given threshold ^3, 4^:

$$UTILITY=1-FPR+q_{P}.(FPR+TPR-1)\;(3)$$

For a convex *ROC* curve in which TPR increases as FPR increases, the derivative of the *Utility* with respect to *q _P_* is zero along the *TPR* = 1 − *FPR* line in *ROC* space ( Figure 1, dashed line). The derivative is negative below this line and positive above it, indicating that points along this line are minima of the *Utility* function with respect to the strategy *q _P_* of the nature player. According to the *minimax* principle, the classifier player should operate at the point along the *TPR* = 1 − *FPR* line that maximizes *Utility*. In ROC space, this condition corresponds to the intersection between a continuous *ROC* curve and the descending diagonal ( Figure 1, empty circle) and yields a *minimax* value of 1 − *FPR* for the *Utility*. It is worth noting that this analysis regarding class skew is also valid for yield/cost skew ^4^.

## Discussion

We showed that binary classifiers may be analyzed in terms of game theory. From the *minimax* principle, we propose a criterion to choose an operating point for the classifier that maximizes robustness against uncertainties in the skew ratio, i.e., in the prevalence of positives in nature and in yield skew, i.e., the yields/costs for true positives, true negatives, false positives and false negatives. This can be of practical value, since these uncertainties are widespread in bioinformatics and clinical applications. However, it should be noted that this strategy assumes that a score optimized for a restricted training set is of general validity. Future studies may apply the minimax principle to optimization of the classifier model and to classifier comparison using other performance measures.

In machine learning theory, *TPR* = 1 − *FPR* is the line of skew-indiference for *Accuracy* as a performance metric ^4^. This is in agreement with the skew-indifference condition imposed by the *minimax* principle from game theory. However, to our knowledge, skew-indifference has not been exploited for optimal threshold estimation. Furthermore, the operating point of a classifier is often chosen by balancing sensitivity and specificity, without reference to the rationale behind ^7^. Our game theory analysis shows that this empirical practice can be understood as a maximization of classifier robustness.
