# Supplementary material for: Optimal threshold estimation for binary classifiers using game theory
Source: F1000Res. 2017 Feb 8;5:ISCB Comm J-2762. Originally published 2016 Nov 25. [Version 3] doi: 10.12688/f1000research.10114.3 (PMC5147524; doi:10.12688/f1000research.10114.3)
Supplement: Peer review history

# Peer review: Referee response for version 3

**DOI:** 10.5256/f1000research.11399.r20025

**Published:** 2017-02-16

**Authors:** Marcel Brun, Arjen ten Have

**Affiliations:** 1 Digital Image Processing Group, School of Engineering, National University of Mar del Plata, Mar del Plata, Argentina; 2 Instituto de Investigaciones Biológicas-CONICET, Universidad Nacional de Mar del Plata, Mar del Plata, Argentina

The authors addressed properly the concerns raised in our previous review.

We have read this submission. We believe that we have an appropriate level of expertise to confirm that it is of an acceptable scientific standard.

# Peer review: Referee response for version 3

**DOI:** 10.5256/f1000research.11399.r20026

**Published:** 2017-02-08

**Authors:** Luis Diambra

**Affiliations:** 1 Centro Regional de Estudios Genómicos, Universidad Nacional de La Plata (UNLP-CONICET), La Plata, Argentina

I have read this submission. I believe that I have an appropriate level of expertise to confirm that it is of an acceptable scientific standard.

# Peer review: Referee response for version 2

**DOI:** 10.5256/f1000research.11243.r18290

**Published:** 2016-12-16

**Authors:** Marcel Brun, Arjen ten Have

**Affiliations:** 1 Digital Image Processing Group, School of Engineering, National University of Mar del Plata, Mar del Plata, Argentina; 2 Instituto de Investigaciones Biológicas-CONICET, Universidad Nacional de Mar del Plata, Mar del Plata, Argentina

The author presents an interesting solution to the problem of selecting the best threshold for a binary classifier which defines a bioinformatics algorithm, when the proportion of positive and negative samples in nature is unknown. His approach consists on using the minimax approach, considering that the goal is to maximize the gain/accuracy (in the specific the case where the yields are 1 for TP and TN, and 0 for FP and FN) in a worst case scenario. For that, the author describes the utility as a function of both the threshold and the nature proportion of negatives q_n.

 Based on the analysis of page 3, given any action of the player (a point in the ROC curve, defined by a threshold), its worst performance (lower utility) will be obtained for some q_n value. The idea is to maximize this minimal, across all possible actions (pairs of FPR,TPR across the ROC curve). This is obtained when the point in the ROC complies with TPR+FPR=1.

 The article is original in the sense that it proposes a valid requirement for the design, to be robust against the unknown state of nature (proportion of positives and negatives), deriving a threshold selection that attains that condition (FPR+TPR=1). Despite the originality of the approach, the article needs minor revisions. In particular it seems the manuscript deals with aspects of pattern recognition from the perspective of Bioinformatics. Although understandable, this is a pattern recognition problem and the work would improve significantly when the role of ROC curves on classifier design, and the mathematics leading to the main result are explained with more detail and improved formalization.

 Below follows a more detailed list of issues that need to be addressed: 

1. The author presents this article in a bioinformatics context that is never specified. For example, in the introduction the authors says "Many bioinformatics algorithms can be understood as binary classifiers, ...", and then refers to "score based binary classifiers". At no part of the article the author provides a list, or some examples, of such algorithms in bioinformatics. Since this is presented as a bioinformatics article, it would be good to include some of such algorithms, which surely were the inspiration for this work. Alternatively, this might be presented in the more mathematical setting of pattern recognition, although this might require more profound changes.
2. Also, it is clear that the problem studied in this article is the one where the classifier model is already defined, producing one ROC curve, and the only adjusting parameter the user has is the threshold. Although this is a rather typical solution in computational biology, it should be mentioned that if the user were able to adjust the classifier, better results might be obtained. There are many references in literature of robust classifier design, some are indicated below.
3. The author should review the statement that ROC curves are often being used for the training of classifiers. The training of classifiers is usually done by minimization or maximization of quality metrics, via the adjustment of the classifier parameters, once the model is defined. Few works are related to training classifiers based on ROC (Some examples are Blockeel, 2002 ^1^ and Srinivasan, 2001 ^2^). On the other hand, classifier evaluation (after training) and model selection are often done via ROC curves. 
  For example, in the second paragraph of the introduction, the author comments that "Binary classifiers are often trained and compared under ... the ROC curve", presenting a reference on the ROC curve, but not to classifier training. Here the term "training" may be confusing, since it is used, usually, for the process of selecting the parameters of the classifiers, from the data, once the model is already selected ^3^. Usually the ROC is used for model selection, where different classifier models are trained (without considering the ROC), and then compared based on their ROC, as described in the reference 2 of the paper.
  Based on the previous comment, the author should also review the sentence, in the same paragraph, where he states "Classifier training often aims at maximizing the area under the ROC". Actually, the training aims, usually, to minimize error, or cost, or other measures of accuracy. Reference 4 of the article, in the first paragraph of the introduction, also makes clear the difference between model building (training), and model evaluation, where the ROC is used.
4. In general, the assumption on a=d=1, c=b=0 could start earlier, simplifying the concepts and equations 1 and 2.
5. The mathematical proof that the minimax threshold is obtained by FPR+TPR=1 requires some elaboration. In one hand, given a classifier trained from data, with a variable threshold t, both FPR and TPR are functions of the threshold, so they would be FPR(t) and TPR(t). From this, the utility is dependent of two variables, q_n and t, so it would be Utility(q_n,t). Now, the problem of finding a minimax value of t would be a bit more complex than the solution described in the article. Still more, one should impose the restriction on the curve (TPR(t),FPR(t)) to be increasing, so that if t2>=t1, then TPR(t2)>=TPR(t1) and FPR(t2)>=TPR(t1). If such condition is held (which is expected on a properly designed ROC curve), then the proof that FPR(t)+FNR(t)=1 is the minimax solution is almost straightforward.
  Without that condition defined above (which is not defined in the paper), one could have a pair (0.8,0.2) which complies with FPR+TPR=1 and (0.81,0.18) that does not comply with that rule, and where the minimum utility (as function of q_n) for the first pair is 0.8, and for the second pair is 0.81, which disagree with the mathematical result presented in the paper. The key here is the increasing nature of the ROC curve. An increasing ROC curve (where TPR increases when FPR increases) could not contain these two points, since an increase in TPR (0.8 to 0.81) should be accompanied by an increase on the FPR (but it goes from 0.2 to 0.18). 
6. Equation 1 uses q_n, which is only defined later, after the equation.
7. The author presents the work in the general context of zero-sum game, with general yield values, but for the main theoretical result, the problem is reduced to the specific situation of a=d=1 and b=c=0. Therefore, the result is not general, only specific to that situation, so it is a little superfluous to maintain all these generic yield values across the paper. For example, Equation 1, when using the costs a=d=1, c=b=0, studied in the theory section, reduces to skew ratio = q_n/q_p, which is more informative for the rest of the paper.
8. In the discussion the author talks about maximizing robustness, but actually the goal is to design "a robust classifier" ^4^. The discussion section also states that the robustness is obtained against a) skew ratio and b) yield skew. This last part (b) is not proved in the paper, since the theoretical results show a minimax results (robustness) against skew ratio, for the specific case of a=d=1 and b=c=0.
  It seems true that no other methods for choosing the operating point present a good rationale beyond mathematical considerations. To strengthen the impact I suggest the author should at least name some of such approaches, like the three ones described by Song, 2014.
9. In a situation of continuous ROC curve, this point should always exist, since it is an increasing curve from (0,0) to (1,1). In the discrete ROC case, which is the usual one when the classifier and the ROC are based on a finite amount of data, this intersection may not occur, and the thresholds may have to be selected using an appropriate interpolation technique. This needs to be clarified.
10. Finally there is a question about the approach. Despite the theoretical attractiveness of the proposed approach, it is not clear how the maximization of expected utility is a desirable quality for a classifier being used in bioinformatics. There is a lot of study and discussion about whether is it better to optimize accuracy, precision, recall, etc, and how each application may require a different goal, but there is not so much discussion about why a classifier should maximize utility in a zero-sum game. The author could address this issue, with a brief statement on the advantages of this measure (the utility) in the context of bioinformatics.

We have read this submission. We believe that we have an appropriate level of expertise to confirm that it is of an acceptable scientific standard, however we have significant reservations, as outlined above.

## References

[1] BlockeelHStruyfJ: Deriving Biased Classifiers for Better ROC Performance. *Slovene Society Informatika*.2002;26(1) :

[2] SrinivasanA: *Machine Learning*.2001;44(3) : 10.1023/A:1010980106294 301-324 10.1023/A:1010980106294

[3] JainAKDuinRPWMaoJ: Statistical Pattern Recognition: A Review. *IEE Transactions on Pattern Analysis and Machine Intelligence*.2000;22(1) :

[4] DoughertyEHuaJXiongZChenY: Optimal robust classifiers. *Pattern Recognition*.2005;38(10) : 10.1016/j.patcog.2005.01.019 1520-1532 10.1016/j.patcog.2005.01.019

# Peer review: Referee response for version 1

**DOI:** 10.5256/f1000research.10895.r17994

**Published:** 2016-12-07

**Authors:** Luis Diambra

**Affiliations:** 1 Centro Regional de Estudios Genómicos, Universidad Nacional de La Plata (UNLP-CONICET), La Plata, Argentina

The author presents a criterion to choose the operating point for a binary classifier. This criterion is analyzed in term of the game theory. By using the mininax principle author proposes to use as classifier threshold the intersection between the ROC curve and the descending diagonal in ROC space. This operating point for the classifier could maximizes the robustness against some bias in the training set. I found some novelty in the fact to consider such bias for an optimal threshold estimation. The paper is well written and organized but I think also that it could be improved by incorporating some general considerations that helping readers to a better understanding of the problem and the present proposition [1,2].

 In the binary classification problem one is trying to deduce the answers to new questions, rather than just recall the answers to old ones. In order to do that we need to train the classifier from question-answer pairs (the training set). This is called supervised learning, because it requires a teacher, knowing the rule, which gives the correct answer to the example questions. In the case here, the author consider score-based binary classifiers, which does not need such learning stage. Could the author put the problem in the context supervised vs. no-supervised?

 In the supervised learning context the classifier threshold is a parameter that is found during the learning stage. Training the classifier maximizing the area under ROC curve is an strategy for the classifier learn the training set. Consequently, the proposed strategy could be considered as a "learning rule". However, the performance over new examples is not guaranteed. Other point which can improve the manuscript would be to consider the ability of generalization of the proposed strategy. Could the author add a discussion in this sense?

 I believe that this manuscript is of an acceptable scientific standard, and that it will be of interest to a wide audience; however, the manuscript could be revised, as outlined above.

I have read this submission. I believe that I have an appropriate level of expertise to confirm that it is of an acceptable scientific standard.

## References

[1] WatkinTRauABiehlM: The statistical mechanics of learning a rule. *Reviews of Modern Physics*.1993;65(2) : 10.1103/RevModPhys.65.499 499-556 10.1103/RevModPhys.65.499

[2] DiambraLFernándezJ: Information theory approach to learning of the perceptron rule. *Phys Rev E Stat Nonlin Soft Matter Phys*.2001;64(4 Pt 2) : 10.1103/PhysRevE.64.046106 046106 10.1103/PhysRevE.64.046106 11690089

# Peer review: Referee response for version 1

**DOI:** 10.5256/f1000research.10895.r17996

**Published:** 2016-12-06

**Authors:** Pieter Meysman

**Affiliations:** 1 Department of Mathematics and Computer Science, University of Antwerp, Edegem, Belgium

The article by Ignacio Enrique Sanchez concerns a common problem in machine learning, namely the selection of the optimal classification threshold, and provides a mathematical solution based on the principles of game theory. The main concern of the article deals with the unknown distribution of positive and negative samples in the ‘real world’ or ’nature', thus beyond the provided training data set. The provided derivation is very elegant, and luckily for those researchers in the field the solutions turns out to be to select a threshold where sensitivity and specificity are equal in the training data set.

 The biggest concern from the perspective of game theory is that ’nature’ is not a conscious agent, and thus will not mischievously choose a positive/negative fraction where the classifier will perform the worst. However as stated in the article, this is to simulate the worst case scenario. However this also means that the threshold calculation may only be optimal in this worst case scenario, but suboptimal in all other cases. It is therefore still not the final word in threshold optimisation, and still leaves machine learning researchers the flexibility to choose other thresholds.

 However I do have a minor comment on the derivation, that I expect can be addressed with small clarifications to the text:

1. The *Accuracy* equals the *Utility* as defined by the payoff matrix in the specific case a=d=1 and b=c=0, which is stated without a loss in generality. However in my understanding, this step makes the assumption that the cost for a false negative and the cost for a false positive is equal, which may not be the case for all classifiers. Thus it is unclear if this specific case can be transposed to all classifiers in general.

I have read this submission. I believe that I have an appropriate level of expertise to confirm that it is of an acceptable scientific standard.
